# “I stayed on the street, as if there was no COVID-19”: enacting practices of care with youth at risk of violence and social exclusion in Portugal

**DOI:** 10.3389/fsoc.2024.1436979

**Published:** 2024-10-28

**Authors:** Patrícia Ferreira

**Affiliations:** Centre for Social Studies, University of Coimbra, Coimbra, Portugal

**Keywords:** COVID-19, care, violence, youth, Portugal

## Abstract

As the COVID-19 pandemic unfolded, a world of carelessness was made visible. Public health guidelines against the spread of COVID-19 (e.g., school closures, staying home, social distancing) have substantially affected youth’s health and well-being and highlighted the need for context-specific understandings of infection, risk, and care. Our research aims to contribute to a deeper understanding of the impacts of COVID-19 on boys and girls serving educational measures and placed in the custody of the juvenile justice system in Portugal during the pandemic. Sustained by the use of ethnographic principles and methods along with participatory techniques, this article uncovers complex entanglements between the public health measures to mitigate contagion and the dynamics that exacerbated socio-spatial dynamics of social exclusion and isolation, educational and (mental) health inequities, and lived and practiced forms of violence by youth-serving tutelary measures in Portugal’s six educational centers. Following Tronto’s feminist ethical–political proposal of care, I argue that COVID-19 became a lens to access youth care needs — self-care, care for others, care as essential work of nurturing affective trajectories and solidarities and promoting positive and non-violent relationships. By caring for youth stories, the engagement of researchers and professionals in this action-oriented research aimed to promote the enactment of practices of care with youth in the Portuguese detention centers as a way to positively affect their inhabited present, promote healthier lives and nurture the construction of caring and non-violent post-pandemic futures.

## Introduction

The COVID-19 pandemic has renewed the debates on the complex relations that connect infectious diseases, the (re)emergence of epidemics, and the systemic conditions that determine their consequences in different contexts and places worldwide. The international response to COVID-19 narrated as a “huge global failure” in the “Lancet Commission on Lessons for the Future from the COVID-19 pandemic” (Sachs et al., 2022), was constructed around scientific and clinical innovations, systemic fragilities, and humanitarian crisis that created and aggravated income loss, unemployment, housing, racial, ethnic, gender inequities and age segregation and discrimination, with even more severe impacts in people with disabilities, mental illness and other infectious and non-communicable diseases.

COVID-19 impacts were not only defined by the lens of the virus, and its long-term effects still remain unclear (Tronto and Fine, 2022). Risk of contagion and illness in communities and populations most affected by COVID-19 were intrinsically connected with socio-territorial inequalities, housing precariousness, and overcrowding (Lages, 2024; Million, 2022), living closer to the poverty line (Schulz et al., 2020; den Broeder et al., 2022), among other sociohistorical, environmental and institutional dimensions that configured the deep production of social, economic, and health inequalities “beyond the direct consequences of the virus itself” (Serapioni and Hespanha, 2023; Ferreira et al., 2020; Souza et al., 2020). While everyday life was reconfigured by the international and national coordination efforts and public health guidelines against the spread of COVID-19, and by new social arrangements, as was the case of lockdowns, isolation protocols, school closures, and social and physical distancing, the “lived experiences and embodied stories and life circumstances” (Filipe, 2024) were often ignored, as was also the case of context-specific configurations of vulnerability (Ford et al., 2023).

As the COVID-19 pandemic unfolded, its profoundly unequal effects made a world of carelessness visible. The United Nations General Assembly has proclaimed October 29, 2023, the International Day of Care and Support^1^. This resolution calls on all agents of society to recognize the importance of care, whose relevance and urgency have been renewed by the COVID-19 crisis. The care provided by healthcare professionals and other essential professionals, but also the care work provided (predominantly by women) for others (children, elderly, vulnerable groups, etc.) has minimized social isolation, replacing fragile health and social support systems in contexts affected by various forms of vulnerability pre-existing or exacerbated by the pandemic (Souza et al., 2020; Linde and González Laya, 2020).

The COVID-19 pandemic is described by Tronto and Fine (2022) as an “acute shock to caregiving around the world,” and that care is not granted in a complex and interconnected world in which “zoogenic pathogens will continue to affect humans around the globe” and “inequalities in both access to and provision of care [will] probably persist.” Care was no longer taken for granted, and the realm of care become more visible as part of individual and collective actions centered on people affected by the new coronavirus, but also by other health problems, forms of vulnerability, and structural conditions that perpetuate diseases and their impacts (Bowlby and Jupp, 2020; den Broeder et al., 2022; Ferreira et al., 2020; Souza et al., 2020), highlighting the need for care to make the world more livable (Puig de la Bellacasa, 2012).

As Joan Tronto proposes, “care is something for which we are collectively responsible” (Parra Jounou and Tronto, 2024) in society, participating in civic life and creating spaces for listening to those who need care, and those who practice care. Care is a disposition, an activity, and a complex and relational process that overflows the public health guidelines disseminated during the COVID-19 crisis. This approach to care calls for a renewed attention to knowledge production in situated contexts where public health and the pandemic lived experiences meet (Ahmad et al., 2020; Ferreira, 2021), and for Berenice Fisher and Joan Tronto’s feminist ethical–political proposal of care:

*“On the most general level, we suggest that caring be viewed as a species activity that includes everything that we do to maintain, continue, and repair our ‘world’ so that we can live in it as well as possible. That world includes our bodies, our selves, and our environment, all of which we seek to interweave in a complex, life-sustaining web (Fisher and Tronto, 1990,40).”*

Within the scope of the call for papers (“Masculinities, nonviolence and empathy”), this work aims to contribute to a deeper understanding of the impacts of COVID-19 on boys and girls at risk of violence and social exclusion placed in the custody of the juvenile justice system in Portugal during the COVID-19 pandemic, and how did COVID-19 ultimately affected *caring with youth* (and also for those who care for them) entering the youth detention centers (YDCs) in Portugal during the pandemic.

As reinforced by the United Nations (2020), the well-being of children and adolescents was substantially affected (emotionally, cognitively, and socially) by the COVID-19 pandemic.

In the section “Research on COVID-19, health and care in YDCs, I start by contextualizing the action-oriented research developed in Portugal under the project “X-MEN: masculinities, empathy and nonviolence.” The methodological approach is framed by ethnographic principles and the use of participatory techniques throughout the research process, and by an ethic of care attentive to our engagement with the participants.

In Results, I provide a contextualization of the pandemic challenges faced the juvenile justice system during the pandemic, how it affected the functioning of the YDCs and a brief portrait of the young people entering the YDCs during the pandemic. In the Discussion, I argue that COVID-19 is a lens to the absence of care practices at the individual, professional, and institutional level, and that its impacts were configured in articulation with the socio-spatial dynamics of social exclusion, violence, and (mental) health inequities. “Caring for stories: COVID-19 as a lens to absences of care” proposes that caring for the youth life stories and listening to the YDCs professionals pandemic narratives are knowledge production practices of “how to care for youth at the YDCs.” engaged. Lastly, in “Enacting practices of care with youth in the YDCs,” I conclude that our engaged research with youth, professionals, and other institutional actors came to nurture the creation of educational and training materials on nonviolence, health, and care sustained the enactment of institutional and professional care practices that promote forms of self and interdependent care (in and outside the YDCs) that may sustain the promotion of youth’ non-violent and healthier post-pandemic futures.

## Methodological framework

### Researching COVID-19, health and care in YDCs

The research presented in this paper is part of the broader research context of the project X-MEN^2^ “Empathy, masculinities and non-violence” concluded in June 2024. X-MEN was financed by the European Union’s Citizens, Equality, Rights and Values programme (2021–2027) under grant agreement 101,049,457. In compliance with the goals of the European Strategy for Gender Equality 2020–2025^3^, the project’s overall objective was to contribute to preventing gender-based violence by addressing the construction of non-violent and caring masculinities with young boys and girls (12–18 years old) at risk of social exclusion in 3 EU countries (Portugal, Spain, Croatia), and whose situation was aggravated by COVID-19 pandemic. The project operated at individual, professional, and institutional levels.

In Portugal, the project’s activities were designed to target a group of young people ‘in conflict with the law’, as is the case of youth-serving tutelary educational measures. The objectives of the X-MEN Portuguese study were, on the one hand, to analyze the COVID-19 impacts on young people’s behaviors and attitudes, as well as on national public policies for child protection and juvenile justice, taking into account the effects of COVID-19 prevention measures on the well-being of youth at risk and on the routines of the YDCs. On the other hand, the project aimed to strengthen the responses of YDCs to the youth care needs by engaging young boys and girls from the project’s initial steps in the production of knowledge concerning the overall project concepts: masculinities, empathy and non-violence. In this article, I focus on the X-MEN research topic “Health, care and COVID-19.” Nevertheless, the analysis of the empirical materials dedicated to this theme is necessarily contextualized in the X-MEN overall contribution to the construction of empathetic, supportive and caring masculinities with the young people who serve tutelary educational measures, and tobreak the cycles of violence that intertwine their life stories. The ethnographic and action-oriented research that sustains this article aims to contribute to a deeper understanding of the impacts of COVID-19 on the lives of 16 boys and girls (12 to 18 years old) serving educational measures in Portugal’s six YDCs subjected to the Contingency Plan drawn up by the Directorate-General for Reintegration and Prison Services during the COVID-19 pandemic (DGRSP, 2020).

The X-MEN approach invokes ethnographic principles and the use of participatory techniques throughout the research process. Our ethical practices implied an exercise of reflexivity and a commitment to fieldwork and the production of knowledge and interventions that respond to the needs of different forms of care (Ferreira and Filipe, 2019).

The X-MEN project was sensitive to the principle of protecting the rights and freedoms of individuals, notably the right to privacy anchored in Directive 95/46/EC (OJEC/23.11.95)^4^ which should be safeguarded about gathering, analyzing, and maintaining empirical data. The research team proceeded according to the national legislation and the Ethics Committee of the Centre for Social Studies (CES), the project host institution in Portugal. Informed Consent was obtained from all participants (legal representatives in the case of minors) participating in this research, and confidentiality and anonymity were guaranteed.

### Data collection and analysis

The study was developed between March and April 2022 in the 6 YDCs in Portugal — two in the North area (Vila do Conde and Porto), one in the Center region (Coimbra), and three in the Center-South area of Portugal (Lisbon metropolitan area). The multi-methods approach consists of quantitative and qualitative methods — a national questionnaire for youth-serving tutelary measures in YDCs, and individual interviews and focus groups with YDCs youth and professionals (Caruso et al., 2023).

The questionnaire provided us with an initial profile of young people serving measures in closed, semi-open and open regimes in the YDCs in Portugal. According to data from the DGRPS, in March 2022 (the month in which the Portuguese team has started fieldwork), 121 adolescents were serving educational measures in closed, semi-open, and open regimes in Portugal, and the questionnaire was applied to 80.16% (corresponding to 97 completed questionnaires) of the universe representative of young people serving tutelary measures in the Portuguese YDCs. The questionnaire was divided into seven thematic topics: sociodemographic data; school path before the YDCs; tutelary educational measures; gender, masculinities and sexuality; health, care and COVID-19; perceptions about the YDCs; culture; sports; youth identity and plans for the future. The questionnaire thematic structure served as a baseline to develop the interviews and focus groups guides. The eleven individual interviews were held with 9 boys and 2 girls in different YDCs to obtain in-depth knowledge about the trajectories, family contexts, educational paths and behaviors that led to deviations from the law. The interviewees were selected in advance by the research team and the YDCs professionals according to boys and girls motivation and willingness to take part in the project’s activities. Each interview was conducted by a member of the research team using a semi-structured interview guide organized into 6 thematic blocks: life and family trajectories; school life before and after entering the YDC; gender relations, masculinities, and violence; YDC and internment measures; health, care, and impacts of COVID-19; life project and prospects. Each interview lasted one hour on average and was audio-recorded with the prior approval of parental or legal guardians, who signed the informed consent form. At the time of the interview, all the interviewees were serving closed educational measure.

Six focus groups were held, one *per* YDC, with boys and girls serving educational measures in open, semi-open, and closed regimes. Each focus group had an average of five participants of varying ages and lasted one hour and a half. The focus groups were spaces for sharing for ideas between young participants who, by listening to each other’s life stories, were able to reflect on their own experiences. By we decided to invite different participants from those interviewed individually to promote the participation of a higher number of young people in the project’s activities. The focus group guide for youth was organized into four thematic areas: knowing each other; masculinities, gender relations, and violence; health, care, and COVID-19; and future perspectives. The aim of the interviews and focus groups with the boys and girls was to promote active listening to their stories — to care for their stories (Ferreira, 2021). By caring for their shared stories, the absences of care that permeate their lives and were complicated by COVID-19 emerged.

In the next step, we organized six focus groups with professionals who work in the country’s six YDCs. The focus groups were approved by the ethics committee and the informed consent was obtained from the participants, audio recorded, transcribed, and analyzed by members of the research team. The focus groups allowed us to access the technical and psychosocial challenges of working in the YDCs during the pandemic. The diversity of interlocutors was fundamental to understanding the professional context as front-line workers facing aggravated technical and psychosocial challenges during the pandemic and the day-to-day life in Portugal’s YDCs. Additionally, these focus groups gave us access to what these professionals consider to be the strengths and weaknesses of their day-to-day work with youth, and the State’s intervention in the juvenile justice system. These professionals are responsible for the day-to-day monitoring of young people who serve educational tutelary measures in open, semi-open, and closed regimes. They are part of the universe of Education Technicians, under the responsibility of the DGRSP, organized into different careers, namely senior social reintegration technicians; professional social reintegration technicians; and other specialized technicians such as psychologists and social workers.

The individual interviews and focus groups created spaces dedicated to listening to YDCs young people and professionals. Together with the additional project’s activities with youth (that will not be explored in this article), these spaces promoted their participation in knowledge production.

Even if this paper will not focus on the subsequent X-MEN actions, it is important to mention that specific he next steps were dedicated to the professionals’ capacity building on X-MEN methodologies, and the promotion of sustainable changes within the institutional contexts, by strengthening the institutional responses and capacity of government officials and decision-makers to promote relations between academia and civil society organizations around GBV prevention programs and inter-institutional cooperation dedicated to the social inclusion of young people affected by violence and social exclusion^5^.

## Results

### The care provided by YDCs during the COVID-19 pandemic

Portugal ratified the Convention on the Rights of the Child (United Nations, 1989) in 1990, and in the following years, the national legal framework underwent significant changes in the juvenile justice model. The Promotion and Protection Law for Children and Young in Danger (LPCJP) and the Educational Guardianship Law (LTE) were approved in 1999, with new changes introduced in 2015 that distinguish the situation of “children in danger, that legitimizes a State’s intervention of protection (LPCJP), from the needs and situation of the children, between 12 and 16 years old, who commit an offense qualified by the penal law as crime and, as a result, justify another kind of intervention, an educational one (LTE)” (Carvalho, 2014). The YDCs are distinguished according to the type of regime — closed, semi-open and open — and educational measures can be applied in the three regimes defined by the LTE, being the closed regime the freedom-depriving educational measure (YDC Padre António Oliveira, Lisbon; YDC Olivais, Coimbra: YDC Santo António, Porto). The YDCs Navarro de Paiva (Lisbon) and Santa Clara (Vila do Conde) are the only ones where girls are held in detention in the three types of regimes. According to the LTE:

“The criteria on which an educational measure is determined by the youth court rely not only on young offenders’ needs, which are evaluated before the sentence by social and psychological or psychiatric assessments but also on the seriousness of the committed offenses in comparison to what is defined in the penal code” (Carvalho, 2014).

These educational measures are implemented by a juvenile justice system that does not have a retributive or punitive purpose:

“It is focused on addressing the offending behavior in a manner appropriate to the young person’s development (Gersão, 2000; Agra and Castro, 2002, 2007). At the core of the LTE is the respect for the young person’s personality, ideological, cultural and religious freedom, within all the rights conferred upon him/her by the Constitution of the Portuguese Republic. Juvenile offenders’ rehabilitation is based on their needs to be educated on the fundamental values for living in society aiming they would assume a constructive role in society as foreseen in Article 40(1) CRC (United Nations, 1989) and in the UN General Comment No. 10 (2007).”^6^ (Carvalho, 2014).

On account of the pandemic situation, the DGRSP produced a Contingency Plan for COVID-19 that determined the measures and procedures aimed at preventing and minimizing the risk of contagion, allowing the functioning of essential activities at YDCs (DGRSP, 2020). The Contingency Plan describes a set of measures focused on the prevention, control, and surveillance of the COVID-19 infection, in which two viruses, SARS-CoV2 and Influenza, were spreading, to reduce the impact of the pandemic on the functioning of each YDC activities. The DGRSP’s Contingency Plan for COVID-19 aimed to prepare and adapt the response of each YDC to the new pandemic context, following a set of measures and procedures to prevent the spread of COVID-19 that included a period of quarantine (14 days) when young people entered/re-entered the YDC, with surveillance for symptoms of COVID-19, and recommendation for isolation in case of presenting symptoms of infection. Also, particular attention was dedicated to the mental health care needs of young inpatients, including medical observation within 24 h, if needed. In addition, entering the YDC required certain procedures as, for example, not sharing personal belongings, cigarettes, clothes, or other items; maintaining good personal hygiene, reinforcing the importance of frequent hand washing; respiratory etiquette; cleaning and tidying up the rooms; adequate use of masks whenever requested. Other specific measures and procedures were to be applied in specific clinical cases, such as flu vaccination and vaccination against *Streptococcus pneumoniae*.

The Contingency Plan was established in each YDC in the country, creating an even greater separation between life inside the detention centers and the surrounding communities, and largely overlooking the risk of devastating consequences from the coronavirus in the juvenile population already known to face significant vulnerabilities [Blum et al., 2019; Ragavan et al., 2020; Comissão de Acompanhamento e Fiscalização dos Centros Educativos (CAFCE), 2021]. The periods of confinement decreed entailed profound changes in the programming and routine of these institutions, with the main measures standing out: suspension of educational activities and sports, cultural and recreational activities that involve going outside or contact with external entities; implementation of alternative strategies for e-learning hampered by insufficient or obsolete computer equipment (implemented as the YDCs had access to the required equipment); suspension of all exits to the outside, except in emergencies; temporary suspension of visits in all YDCs; extension of summer vacations; modifications to the use of YDCs spaces by the circulation, distancing, and capacity rules imposed by the Directorate-General for Health; authorization for video calls and extension of the number and duration of phone calls; creation of specific units (located in the YDC Santa Clara and YDC Bela Vista) for prophylactic isolation; implementation of an Internal Vaccination Plan against COVID-19 (starting in January 2021).

Given the evolution of the pandemic from 2020 to 2021, an increased number of young people became infected with COVID-19, as well as Professional Social Reintegration Technicians (TPRS). If the number of TPRS in the YDCs was already insufficient, prophylactic isolation and infection have worsened this situation (Caruso et al., 2023, p. 54–55, table 10 and graph 4). In light of the evolution of the pandemic and the implementation of the national vaccination plan against COVID-19, it was possible, since the second semester of 2021, to ease the restrictions imposed for public health reasons, with the Committee for the Monitoring and Supervision of Educational Centers (CAFCE) reinforcing as the main deconfinement measure the resumption of school activities and professional training in a face-to-face format, return to face-to-face visits, the scheduling of vacation periods so that young people would simultaneously carry out the quarantine period on their return to the YDCs, and the progressive opening to the presence of external entities (e.g., art development program) and the reopening of outdoor activities.

In an assessment of the YDC’ response to the effects of the pandemic between 2020 and 2021, Comissão de Acompanhamento e Fiscalização dos Centros Educativos (CAFCE) (2021) highlights the rapid reorganization of services, which contributed to a reduced number of infected young people, as well as the flexibility and adaptability of the YDCs to mitigate the effects of the country’s two major lockdown periods by reinventing the activities under the circumstances, while maintaining some routines and the necessary structure of the daily lives of young people in detention, which has contributed to the positive adherence by young people to the changes in routines imposed by lockdowns. Nevertheless, it is important to recognize the difficulties faced during the process, including the delay in resuming virtual school activities due to the late delivery of the necessary computer equipment, as well as the burnout of professionals, with tough schedules and subject to long periods away from their families and, in a subsequent phase, the difficulties of the young people in staying motivated and complying with the YDC routines in face of prolonged experiences of isolation and solitary confinement (Gagnon, 2020), which was especially harmful for children and youth (Barnert, 2020). The lack of investment in the mental health of young people in detention through the provision of systematic psychological and psychiatric care was also detrimental since the juvenile justice population has profound mental health morbidity, often related to previous trauma, that was exacerbated during the pandemic by fear, social distancing, and disruptions in care, housing, schooling, and daily routines.

As the Working Group that presented the Proposal for a Health Plan in the Context of Deprivation of Liberty 2023–2030 (2023) points out, admission to the justice system is often the first time these youth has access to health care. Thus, the YDCs are often the space that respond to health situations that have not yet been addressed. However, because of the circumstances in which these young people are place, they can also be spaces that induce mental illness and create health inequalities.

### Outside, on the street

As the periods of compulsory confinement were imposed between 2020 and 2021, people were expected to restrict themselves to the space of their homes. Yet, the social and economic vulnerability and house precariousness conditioned staying at home during the imposed lockdowns (Lages, 2024). In Santo António YDC, Porto (April 2022), a recurring theme in the focus group was “the father’s return home, after serving a prison sentence and the recurrent episodes of domestic violence experienced by the mother and her children.” The childhood paths and juvenile life trajectories are composed of accumulated and recurrent violence.

Concerning their territorial belonging (place and type of housing), more than half of the respondents to X-MEN questionnaire (53%) live in social housing, and mention the socio-spatial stigma associated with poverty, low income, precariousness, crime, and violence. The respondents mentioned that, outside the YDCs, discrimination and prejudice (experienced by them and practiced on others) are also frequent concerns, related to the place where they live (24%) and the color of their skin (11%), i.e., territorial and ethnic-racial discrimination (Afro-descendants and Roma).

The questionnaire also revealed that the pandemic exposed the socio-economic and, in particular, housing constraints under which they lived. Their houses were too small for the number of inhabitants living in them and, as consecutive periods of compulsory confinement were imposed, they were expected to restrict themselves to the space of their homes. Thus, most of the youngsters report not having complied with the obligation of staying at home, and the conditions for being on the streets at serious safety risk were created (Lages, 2024; Bowlby and Jupp, 2020; Cohen and Bosk, 2020).

Most of the young people that participated in the questionnaire were born in Portugal, other countries of birth are mentioned in smaller numbers, namely Brazil and Portuguese-speaking African countries such as Cape Verde, Angola, and Mozambique. It is the case of Filipe^7^, 16 years old. In the interview, he shared that he is Brazilian and came to Portugal in 2019 with the help of his father, who was already living here. His daily memories of homicides, drug trafficking, and theft in Brazil are still very present. Even though he felt abandoned by his father since his childhood, it was he who “freed” him from the announced death by gangs in Brazil. Filipe had already been detained three times in Brazil and, from a very early age, he was already in the police’s sight. In Portugal, he started working on construction sites with his father. However, conflicts with his stepmother, the one whom he believes was responsible for his parent’s separation, and his father’s abusive use of alcohol, made living at home increasingly tense. Three months later, the pandemic happened. Even with the public health restrictions, Filipe started to go out in Lisbon, made new friends, and, in a short time, committed robberies that resulted in educational tutelary measures. Aggression, in his view, is committed by the police and not by him and his friends, referring to police vigilance that targeted them during the pandemic. By going to the empty streets, Filipe and his friends became more exposed to police control. Other participants also mentioned were subject to frequent raids in which they were repeatedly searched by the police, reporting cases of beatings, intentional non-use of identification on the part of law enforcement agents, among other practices that led them to look with permanent suspicion towards police authorities. As a result, most young people reported not having complied with the obligation to stay home and stayed on the streets, being the ones present in public spaces (specifically the boys).

Dinis is Portuguese and is about to turn 18. He is serving tutelary educational measures at the Olivais YDC for over two years. During the interview, he also tells us about his experience with the police, saying that at one point, when he was caught and taken to the police station, the officers did not realize that he was a minor so they beat him, both on the street and inside the police unit. They only knew his age when they took him back to his mother’s house. The violent events he suffered and practiced in the schools he attended and on the street, as well as the situations experienced during police raids, are recounted by Dinis with a certain ‘tranquillity’ as if they were part of his life. For this reason, he emphasizes that if he were on the streets, he would probably be on the same path.

The cycles of violence these adolescents have already lived contribute to health risk behaviors, like the use of drugs. Diogo started using pot and hashish at the age of 9 and is now 18 years old. In the interview, Diogo says that he is violent and impulsive, and has a hard time controlling his anger. He cannot stand people talking loudly to him, and he has already hit a teacher. From the age of 6 to 7, he was put on medication, but he felt that he was becoming dependent on it, as it was a powerful medication. At the Santa Clara YDC, he is medicated to sleep and to “control his impulses,” as he puts it, but not in the same way as before, so he feels better.

The story told by Matilde (17 years old) during the interview is marked by her time in foster homes from the beginning of her teenage years. Matilde tells us about her i relationship with drugs, which is why she sees the YDC as a place that keeps her away from addiction.

*“[…lost] trapped in drugs. I may be a girl, but I’ve had a lot of experience with it and I’ve used everything you can imagine. The only thing I have not done is drug injection, I do not want to and I do not intend to, but I’ve been involved in a lot of things. My mother has no idea, nor do my parents, I’m a bit ashamed of that and I did not want to tell my parents. My parents had already seen me in a critical state—oh, I’ve seen you like that and I do not want that for you—and that hurt me, hearing those words from my mother, saying that she does not want that for me. But I’m going to let it go. I do not have the willpower on my own, I need help and that’s why I came to the YDC. The doctor said that I’m going to start with consultations, she’s already made an appointment, I’m going to start with support consultations for drug addicts to see if, when I go outside, I can help myself.”*

For Matilde, “falling apart” meant conflicts with her mother, regular beatings, a lack of interest in school, failing grades, and drug use. This scenario of permanent conflict resulted in her mother’s decision to send her to a foster home. Matilde is in the YDC for theft. However, throughout her interview, she describes the YDC as a rehabilitation space for drug addicts. She shares that she has suffered violence from her boyfriend, but she attributes his aggression to the effects of the drug use. She often recounts her mother’s violence against her and how this context of physical aggression was the defining factor that determined having lived in foster care. While Matilde describes this period of her life with sadness, she also stresses that the YDC has freed her, guaranteeing a “cure” for her drug addiction, finishing school, and the possibility of leaving with a profession. She knows it will be difficult to conclude her studies outside the YDC because she will be over 18 by at the time she leaves, but she has hope for her future.

It was noteworthy that, in some cases, the participants declared that they “worked in drug trafficking” or theft. Before being subjected to the more severe tutelary measures, they were previously subjected to less severe measures, such as educational monitoring. 82% of the surveyed are currently serving a closed educational measure, the most restrictive according to Portuguese Law, based on acts analogous to crimes of a serious nature. Manuel shares that there may even be drugs in his neighborhood, but this did not influence his conduct. He explains to us that he has been involved in gang fighting, and highlights the similarities with the trajectories of his cousins: thefts, their love of football, and their time in YDCs.

COVID-19 materialized an increased risk of exposure to violence and detention, but it wasn’t perceived as a health risk outside the YDCs, on the street. Between February and March 2022, more than 2 years after the start of the COVID-19 pandemic, 39% of respondents said they had had the disease, while 52% had not and 9% did not know if they did. As for their family members, they said 78% of them had been infected with COVID-19. The high number of infected relatives fortunately did not translate into deaths, as only 5% of the young men and women interviewed reported having relatives who had died as a result of the virus. However, knowing whether or not they had had the disease was not enough to predict the impacts of this on their life dynamics. Most of the interviewees state, as we see above, only began to understand the dimensions of the pandemic in all spheres of social life when they entered the YDCs. Only at this moment did they have contact with quarantine protocols, social isolation, suspension of visits, vaccination, among other implemented health and educational measures.

### Inside the YDCs

According to the YDCs professionals, the lack of basic health care throughout the youngsters’ lives, the fact that many of these boys and girls only begin to receive regular medical care for the first time in the YDCs, the lack of hygiene habits, of sleeping or eating schedules, the significant use of alcohol and smoking (22%), and drug use (49% of the surveyed reported using drugs such as weed, marijuana, hashish, and cocaine every day) and the continuous use of anxiety-controlling medication (4%) relate to their “unruly lives” outside the YDCs (Caruso et al., 2023, p. 67). Oral health appears as the major health issue in the professionals’ assessment as an “epidemic of poor dental health” that characterizes this population with impact on their self-perception, and even on stigmatization. For these reasons, oral hygiene habits are promoted inside the YDCs:

*“Outside the YDC they did not practice oral hygiene and now they do it 3/4 times a day” (Specialist technician, focus group, Olivais YDC)*.

Poor eyesight is also mentioned as a neglected health issue, followed by what they call “emotional issues,” the affective needs and mental health challenges they face from an early age, and were accented during the implementation of the YDCs Contingency Plans. The transition to the YDCs is a difficult process for youth, since they leave home, friends, etc., and psychiatric medication can be useful during this process. Most young people have regular child psychiatry appointments (once a month). Some were already under medical supervision before entering the YDC and continued to be monitored by their doctors. Mental health issues are a matter of care for the YDCs professionals, and they are often pre-existing to youth arrival at the YDCs. It was significant to note that 60% of the respondents take medication for mental health issues regularly, such as depressive disorders, emotional instability, anxiety, and Attention Deficit Hyperactivity Disorder As represented in the word cloud in the X-MEN final report research by Caruso et al. (2023, p.69), youth in detention can identify the names of the medicines they take and their properties, namely antidepressants, antipsychotic, and anxiolytic drugs. When they did not know the name for sure, they simply answered: “to sleep.” Others mention taking supplements: “I take it here, this is not even medicine, it’s something to help me sleep, it’s Melamil” (Manuel, 16).

Many of the young people narrated they began to feel better about their bodies after entering the YDCs due to the new health habits they acquired, sometimes for some for the first time in their lives. Adopting self-care routines inside the YDCs is a strategy for strengthening health, and self-esteem, and a ‘gateway’ to develop sel-care. Regular physical exercise, healthy diet (some of them reported being overweight because of their poor eating habits outside the YDCs), regular hygiene, and sleeping habits are reinforced in the lives of these young people from the moment they enter the YDCs. Filipe says that he now goes to the gym everyday, and to the psychology appointment every week, on Thursdays. Dinis shared that from an early age he had always “been around psychology,” but “psychology did not result in anything.” Even if he does not value the psychological support, he appreciates the support given by the YDCs technicians, and even the security guards, who are present everyday in his life. As the professionals summarize the routine of the YDCs, there is a rhythm of functioning, paced, and controlled to guarantee that young people internalize habits and rules. A normal day for a youngster in a YDC usually follows a schedule: being alone for 10 consecutive hours; being in classes and/or vocational training for 8 h with short breaks (mid-morning and mid-afternoon) and the lunch break; carrying out cleaning duties for 1 h a day; the remaining hours are spent in activities related with the two daily assessment moments with the YDC professionals (late morning and late afternoon), in addition to relaxation periods during meals, the use of the games room or the sports field.

Once inside the YDCs, social isolation required as a measure to protect oneself and others was considered the first and main impact of COVID-19. Most of the respondents shared that they only began to understand the importance of the pandemic when they entered the YDCs in 2020, an event that brought them to a standstill. When he arrived at the YDC Santo António, in Porto, for the 14-day quarantine period, Manuel says it was “the worst moment he’s ever been through,” and “his head told him to give up.” “The penny dropped” for Manuel. Before entering the YDC, he told us, “I stayed on the street as if there was no pandemic.” At the beginning of quarantine, Manuel felt really sad:

*“I could not be with anyone, without a mobile phone, without anything, isolated without anything, I even wanted to kill myself, in my head it even happened, but after three days I could leave the room to watch television, and then I was calmer.”*

The loss of freedom and the imposed confinement were aggravated by isolation before even knowing in which YDC he would serve the determined educational measures. Would it be close to family? Would it be far from home? In Manuel’s case, the distance of more than 200 km between Amadora and Coimbra meant that, over six months, he only received two visits, one from his grandmother, and the other from his mother. These interruptions in Manuel’s life affected his mental health. Before entering the YDC, “I kept in the street as if there was no pandemic,” Manuel told us. Inside the YDC, he began to take medication to sleep. The COVID-19 pandemic has translated into a sentence of isolation marked by the absence of physical and social contact (“we could not give our family a hug”) since the restrictions imposed have led to the impossibility of visits from family or friends, and no trips home.

Inside the YDCs, COVID-19 was configured as a risk to health. The implementation of sanitary protocols, social distancing, suspension of visits, and vaccination, the conversion of all tutelary measures into educational measures carried out in a closed regime, among other measures included in the Contingency Plan, created the feeling that the ‘world has stopped’ with COVID-19.

Adapting to new rules, the pace of online learning, cultural and regional differences, and the distance from family and home was only eased by the accompaniment of the YDC professionals who, in addition to their professional responsibilities, often appear in the interviewees’ imagination as the maternal and paternal figures that were absent in the course of their lives. COVID-19 posed a new challenge to the health and safety of vulnerable youth, but COVID-19 impacts are not only defined through the lens of infection (Cohen and Bosk, 2020). Its consequences on the lives of young people, both inside and outside the YDCs, have affected mental health, well-being, educational paths, relationships, and exposure to violence in the social and spatial contexts in which they live and which have placed them in YDCs during the pandemic.

With a long journey to complete, talking about a post-COVID life means talking about life outside, about a future that appears distant and uncertain, in contrast with the difficult and lengthy process of adaptation to the functioning of YDCs during the pandemic. COVID-19 has made it harder to ensure continued intervention in health promotion, access to adequate healthcare, and to provide the necessary attention to the multiple health care needs since the control measures to stop the spread of COVID-19 were urgent. As Barnert (2020) recalls, responding to these care needs calls “for pursuing alternatives to incarceration, delivery of clinical care and support services equal in quality to standards applied to all children, and attentiveness to the unique needs and vulnerabilities of children impacted by the justice system.” Nevertheless, Filipe had a pleasant surprise when he found out that the YDC wasn’t as bad as he had imagined: “It’s like a hotel here,” “I went back to school here,” “There’s even a swimming pool here,” in opposition to the images of bars, high walls, and high wire he had imagined.

The 24-h routine of the YDCs professionals who observe, plan, evaluate, and control is entangled with emotions, conflicts, human and technical needs that result in strenuous work, also dictated by the pace of schedules, stress, and low salaries. Additionally, during the pandemic they were unable to be confined at home with their families since they were front-line workers with a high risk of exposure to the virus. In the focus groups, they relate mental suffering, fatigue, burnout, stress, exhaustion, poor food schedules, insomnia, depression, and anxiety and the need for medication to help them with these mental health issues.

Socio-affective involvement with the youth is indisputable, and the professionals taking part in the study made a point of highlighting how much this “profession takes a lot of effort”:

“This is a very stressful profession. It’s tough and exhausting trying to be a 15- year-old, to follow the thoughts of a 15-year-old and then come back to yourself” (Professional Technician in Social Reintegration, male—YDC Santa Clara).

One professional also pointed out that:

“[t]he kids are very attached to us and with the pandemic, having to move away from physical contact made it very difficult for them” (− Professional Technician in Social Reintegration, YDC Santo António, Porto).

Another colleague added that they “live in a state of constant alert” since they are dealing with highly structured and hierarchical environments, regulated by rigid and detailed rules regarding every aspect of the life of every young person who enters YDCs. In addition to their impacts on young people’s lives, these professionals are also overwhelmed by the need to constantly monitor them, manage their daily lives, take care of their needs, prevent any conflicts from escalating, and anticipate problems. Their work is to ensure that these young people will leave the YDCs with a project for their future that gives them a real chance of a non-violent future and fulfilling their potential. To this end, the YDCs try, albeit with serious constraints, to diversify their training offer, liaising with the local community to guarantee work for these young people, according to their specific needs. However, they recognize the need to work with the external context to the YDCs, namely with the families and community networks to which the young people will return. This reinforces some of the young people’s concerns and fears about the future since, as one YDC professional recognizes, “Nobody wants these youngsters back” (Professional Technician in Social Reintegration, YDC Santo António, Porto).

## Discussion

### Caring for stories: COVID-19 as a lens to absences of care

Our research highlighted some of the complex entanglements between the compulsory public health measures to mitigate contagion, and its impacts on the lives of vulnerable youth that entered the YDCs during the pandemic. COVID-19 was less perceived as a risk to health as an infection when facing other absences of care that composed youth’s trajectories during the pandemic. COVID-19 has uncovered various asymmetries within the YDCs, particularly in the lack of resources to guarantee the right to health for all young people. The contingency plan applied in the YDCs included prophylactic measures such as an initial quarantine period, and the conversion of all internment measures into a closed regime translated into a sentence of isolation for young people adjusting to the functioning of these institutions. As the work carried out by other authors in different parts of the world support, social isolation, economic stressors, and fear of the virus contributed to increased rates of anxiety, depression, sleep disorders, and other mental health issues particularly among young people from vulnerable communities, or living under social and institutional care (Barnert, 2020; M’Jid, 2020; Ng and Ng, 2022; Santomauro et al., 2021). Also, mental health risks in youth grappling with aggression and multiple forms of violence were aggravated by the (un)expected consequences of public health guidelines implementation in the contextual socio-special dynamics that restricted them to unsafe environments, such as abusive households, housing insecurity, or increased exposure to violence on the street (either through the practice of violence or greater exposure to police surveillance). It also limited their access to support systems outside the home, such as child protective services, educational, mental health support and counseling services, and medical services provided in institutional settings, and created the idea that the ‘world has stopped’ with COVID-19 (Nadeem and Van Meter, 2023; Chiesa et al., 2021; Ragavan et al., 2020; Caruso et al., 2023).

In my previous research focused on collaborative knowledge production and the creation of care practices in the context of epidemiological and social research on infectious diseases in Brazil, I became aware of the importance of supporting the creation of spaces for caring for the stories of those who are affected by health problems and structural vulnerability and need diverse forms of care (Ferreira, 2021). Care, in this context, is “the commitment to the future of those who will be most affected by the epidemic [and] is guided by a concern with those who care” (Ferreira et al., 2020).

By caring for the “stories of suffering, violence, social exclusion and complex entanglements between health, disease and the social processes that compose ways of living with infections and structural vulnerability” (Ferreira, 2021, p.18) we access absences of care that need our care. This sustained our commitment to take the next step to focus on the practices of care with youth in the YDCs from the perspective of “caring with,” the fifth phase of care proposed by Tronto (2013), in which caring needs are also consistent with democratic commitments and actions towards justice and equality.

### Enacting practices of care with youth in the YDCs

As Becerra and Muneri-Wangari (2021) say, “the COVID-19 virus has been a trigger for emerging practices of care by being an actor with agency that transforms the everyday life of subjects by placing them under uncertainty.” Not only in the YDCs, COVID-19 led us to the uncertainties of living in anticipation of future crisis and how they will impact vulnerable groups and individuals who are also the ones who feel the consequences of unequal processes most acutely and experience their effects for a long time (Sachs et al., 2022).

In the focus group, the YDCs professionals recalled that part of their work with youth is to help them define a plan for their future outside the YDCs. The affective bonds they establish with youth is a care practice, and this is relevant since the youngsters highly value their relationships with the YDCs professionals, and their openness to listening to them (something they often did not have at school, or in the community and family contexts). They also recognize the need to work on the external context, namely families and community networks to which young people will return after completing their detention measures. These caring relations are recognized as driving forces of positive behavior, interdependent care (in and outside the YDCs), and the promotion of youth’ non-violent and healthier futures once outside the YDCs. In the context of X-MEN research, the engagement of youngsters and professionals in reflexive and participatory actions approaching topics such as COVID-19, self-care, non-violence, caring masculinities (Elliott, 2016), mental health and medicalization, healthy relationships at the individual and group levels became vital to support knowledge production and interventions dedicated to the professionals training and public policies recommendations on these matters. As Tronto (2010) says, “good care in an institutional context has three central foci: the purpose of care, a recognition of power relations, and the need for pluralistic, particular tailoring of care to meet individuals’ needs.” By orienting knowledge production to the creation and promotion of caring institutional and professional care practices in the YDCs it is our objective to contribute to provide safe and supportive environments for youth to flourish, strengthen social support networks, address systemic issues such as social, health, and gender inequalities. This was implemented in the subsequent project phases by developing collaboration efforts with government agencies and political stakeholders (Langford et al., 2017; Laurin and Martin, 2022; Tronto, 2010). As Tronto says, “Care is something for which we are collectively responsible. As a consequence, a revolution in institutions and practices of care will require a parallel revolution in political and social institutions and practices” (Parra Jounou and Tronto, 2024).

## Conclusion

This research aims to highlight the complex entanglements between COVID-19, the public health measures to mitigate contagion, and the dynamics that exacerbated social exclusion, precariousness, and lived and practiced forms of violence of youth-serving tutelary measures in Portugal’s six YDCs. It contributes to a deeper understanding of the COVID-19 impacts as not being only defined by the lens of infection or health risks, towards more complex understandings of COVID-19 as (an)other matter of care entangled with other (mental) health concerns, social exclusion, vulnerability, and lived and practiced forms of violence within the social and spatial contexts they inhabit.

Following Tronto’s theoretical and ethical proposal of care, our work opened new public health and social research perspectives on care in a post-pandemic world. The X-MEN research on health, care, and COVID-19 was guided by caring for the stories of those who need care and those who care. The intervention-oriented knowledge production sustained the enactement of practices of care with youth by the institutional and professional actors dedicated to their care, positively affecting their inhabited present, and nurturing the construction of possibilities of youth’s non-violent and healthier futures.

## Data Availability

The raw data supporting the conclusions of this article will be made available by the authors, without undue reservation.

## Footnotes

^1^ https://www.un.org/en/observances/care-and-support-day

^2^ https://xmen.ces.uc.pt/

^3^ https://ec.europa.eu/newsroom/just/items/682425/en

^4^ https://eur-lex.europa.eu/eli/dir/1995/46/oj

^5^“Online training for professionals working with youth at risk: Generating Empathy, Non-Violence and Gender Sensitive Interventions” and “Recommendations for Public Policies Targeting Youth at Risk In Portugal, Spain, and Croatia” available at https://xmen.ces.uc.pt/resource_country/portugal/.

^6^For a debate on the need to improve juvenile justice practices in Portugal and the public debate on the implementation of a ‘child rights perspective’ in all the national policies related to childhood and youth, see Caruso et al. (2023).

^7^We have chosen to use fictitious names to protect the identity of the interviewees.
